# Multi-Feature Re-Identification Enhanced Dual Motion Modeling for Multi Small-Object Tracking

**DOI:** 10.3390/s25185732

**Published:** 2025-09-14

**Authors:** Ruiqi Ma, Qinghua Sheng, Yulu Chen, Zehao Tao, Sheng Wang, Xiaoyan Niu, Shuhan Chen

**Affiliations:** 1 The School of Electronics and Information, Hangzhou Dianzi University, Hangzhou 310018, China; mrq1207@hdu.edu.cn (R.M.); sheng7@hdu.edu.cn (Q.S.); tao_zehao@hdu.edu.cn (Z.T.); wang_sheng@hdu.edu.cn (S.W.); 2 Zhejiang Key Laboratory of Intelligent Vehicle Electronics Research, Hangzhou 310018, China; 3 Institute of Advanced Magnetic Materials, College of Materials and Environmental Engineering, Hangzhou Dianzi University, Hangzhou 310012, China; chenyulu@hdu.edu.cn; 4 The College of Electrical Engineering, Zhejiang University, Hangzhou 310027, China; 11410057@zju.edu.cn

**Keywords:** small-target tracking, multi-object tracking, Kalman filtering, optical flow, multi-feature fusion

## Abstract

Multi Small-Object Tracking (MSOT) is crucial for drone inspection and intelligent monitoring, yet traditional Multiple-object Tracking (MOT) methods perform poorly in such scenarios. The reasons include the following: small targets have low resolution and sparse features, leading to high missed detection rates; frequent occlusion and motion blur in dense scenes cause trajectory interruption and identity switches. To address these issues, an MSOT method combining dual motion modeling and dynamic Region of Interest (ROI) detection is proposed. The dual motion framework integrates Kalman filtering and optical flow through dynamic weighting to optimize target state estimation. The Kalman filter-guided dynamic ROI mechanism, combined with multi-feature fusion, enables trajectory recovery when targets are lost. Experiments on the VisDrone-MOT and UAVDT datasets show that this method outperforms mainstream algorithms in core metrics such as MOTA and HOTA, with better trajectory continuity and identity consistency while maintaining good real-time performance.

## 1. Introduction

Multiple-object Tracking (MOT) is a core task in computer vision, which detects and continuously tracks multiple targets in video sequences while maintaining their consistent identities. With the development of technologies such as autonomous driving, intelligent monitoring, and drone inspection, the demand for MOT in complex scenarios is growing day by day.

Based on the integration mode of tasks, MOT is mainly divided into two categories: Two-Stage Multi-Object Tracking (TSMOT) and joint detection and tracking (JDT) algorithms, which differ significantly in technical implementation and application scenarios. Two-stage multi-target tracking includes two independent stages: detection and association. In the detection stage, a target detector locates targets frame by frame and extracts appearance features. In the association stage, data association algorithms are used to match detection boxes from adjacent frames. The joint detection and tracking algorithm realizes in-depth collaboration between detection and tracking and adopts an end-to-end architecture.

However, Multi Small-Object Tracking (MSOT) faces unique challenges, which manifest in three aspects: First, challenges from the targets themselves. For instance, long-distance pedestrians, infrared targets, and small targets in drone aerial imagery have low resolution and sparse texture features, making detection and matching much more difficult. Second, the impact of background interference. Complex backgrounds can further obscure the feature information of small targets, reducing tracking stability. Third, dynamic interactions in dense scenes. Targets are often occluded and cross paths, which directly breaks the trajectory continuity of traditional tracking methods and leads to significant performance degradation.

Traditional MOT methods have been developed in two major paradigms: statistical filtering-based and computer vision-based methods. Statistical methods, such as Multiple Hypothesis Tracking (MHT) [1] and Joint Probabilistic Data Association (JPDA) [2], excel in handling data ambiguity but struggle with small targets—this is because they rely on high-quality point measurements (e.g., precise position coordinates), which are scarce for low-resolution small targets. Random finite set (RFS) [3] models target sets as random variables to handle unknown target counts, but their former high computational complexity issue has been alleviated by advances like the Labeled Multi-Bernoulli (LMB) filter [4]—which decomposes multi-object density into independent labeled Bernoulli components for linear-complexity data association and efficient SMC implementation. However, in dense small-target scenarios (e.g., crowded aerial tracking), the interaction-aware LMB filter still faces challenges: fine-grained state corrections for target interactions and distinguishing overlapping sparse-feature targets offsets complexity gains, so the real-time constraint remains valid here. With the rise of deep learning, computer vision-based MOT (e.g., two-stage TSMOT and end-to-end JDT) has become mainstream for small-target tracking. Even so, computer vision-based methods tailored for small-target MOT still have three key limitations: For two-stage TSMOT methods (e.g., SORT [5], DeepSORT [6]), they rely on Kalman filtering for motion prediction, but the linear motion assumption fails in fast-moving or occluded small-target scenarios. For end-to-end JDT methods (e.g., TraDeS [7], OffsetNet [8]), while they reduce detection error accumulation, they often sacrifice real-time performance due to complex feature fusion. For small-target-specific trackers (e.g., DroneMOT [9]), though they improve detection accuracy, they lack adaptive recovery mechanisms for temporarily lost targets. To tackle these issues, this article proposes solutions from two aspects: motion modeling and trajectory recovery.

Dual motion modeling framework: It focuses on two key challenges; one is the large trajectory prediction bias of traditional Kalman filtering in occluded scenes, and the other is the high computational complexity of optical flow methods. By integrating two types of motion features, a new model is constructed. This framework uses dynamic weighting to combine the global linear prediction of Kalman filters with the local pixel-level motion capture of optical flow features, breaking the adaptation limitations of a single model in complex motion scenes. Compared with methods relying solely on Kalman filters (such as SORT [5]), it significantly improves the tracking stability of motion-blurred targets.

Local detection enhancement mechanism: It aims to solve trajectory interruption caused by missed detection of small targets and severe occlusion. Its core lies in targeted processing of target disappearance positions: it leverages Kalman filtering to guide the generation of dynamic Regions of Interest (ROIs) and combines multi-feature fusion for re-detection within these regions, thereby achieving target repositioning and trajectory continuation when the detection algorithm fails.

Core contributions of this article include the following:

- A dual motion modeling framework is proposed, which integrates Kalman filtering and optical flow features. It addresses the performance bottleneck of traditional single-motion models in occlusion and motion blur scenarios. A dynamic weighting mechanism is used to integrate the global linear motion prediction of Kalman filtering with the local pixel-level motion information of optical flow features. This balances the long-term stability of uniform velocity models and their ability to respond to sudden motion changes. It optimizes the accuracy of target state estimation, effectively reduces prediction lag caused by intense motion or occlusion, and improves tracking robustness in occlusion and motion blur scenarios.
- A local detection enhancement mechanism is designed. It is based on a Kalman filter-guided dynamic ROI detection strategy. This addresses trajectory interruption caused by missed detection of small targets and severe occlusion. When a target is temporarily lost, a local ROI region is dynamically generated based on the predicted state of Kalman filtering. The search scale coefficient is adaptively adjusted according to historical trajectory speed and confidence. Re-detection is then performed within this region. This balances search efficiency and the risk of missed detection. It significantly improves the detection success rate in scenarios involving missed detection of small targets and severe occlusion. It also realizes the reconstruction and tracking recovery of lost targets and enhances trajectory continuity.
- A multi-feature fusion target association mechanism is developed. It integrates appearance, geometric, and motion information. This enhances the robustness of target re-identification in complex scenarios. HSV color histograms (with Bhattacharyya distance as the metric), geometric features (aspect ratio and area constraints), and motion information (Kalman predictions) are combined. These perform multidimensional validation of candidate regions. This enables accurate target re-identification and consistent ID maintenance when targets are occluded or temporarily lost. It further ensures trajectory continuity and identity consistency.

Compared with traditional methods, this approach significantly improves tracking robustness and trajectory continuity in scenarios involving small targets and dense occlusion.

## 2. Related Work

### 2.1. Two-Stage Multi-Object Tracking

The two-stage multi-target tracking algorithm decomposes the entire tracking process into two independent stages—detection and association—where each stage undertakes distinct responsibilities and works in concert to accomplish the target tracking task.

In the detection phase, the core task is to use an object detector to locate targets frame by frame and extract their appearance features. In the early days, two-stage detectors (e.g., the R-CNN [10] series) dominated. These detectors generate a large number of candidate boxes via region proposal networks, followed by classification and regression of these candidate boxes. While this method offers high detection accuracy, it suffers from high computational complexity and poor real-time performance. With technological advancements, single-stage detectors—represented by the YOLO [11,12,13] series—have become the mainstream choice, thanks to their speed and efficiency. Treating object detection as a regression task, YOLO-series detectors directly predict object categories and positions on feature maps, greatly boosting detection speed. Meanwhile, detectors like Faster R-CNN [14] and SSD [15] play important roles in different scenarios, each with unique advantages suited to scenarios with varying accuracy and speed requirements.

In the association stage, the algorithm needs to match detection boxes across consecutive frames to form complete target trajectories. This process is typically accomplished via data association algorithms, with the Hungarian algorithm and Kalman filter [16] being widely adopted. The Hungarian algorithm solves optimal matching problems, while the Kalman filter models the target’s motion state to predict its position in the next frame. By combining these two, correlation costs are calculated based on Mahalanobis distance and appearance similarity, enabling effective matching of detection boxes between adjacent frames. Take the SORT algorithm [5], for example: it uses the Kalman filter for motion prediction and integrates it with the Hungarian algorithm to achieve efficient matching. Its independence from target appearance features gives it excellent real-time performance, making it particularly suitable for resource-constrained scenarios. However, relying solely on motion information, this algorithm is prone to issues such as target ID switching and trajectory interruption in scenarios involving occlusion, dense target interaction, small target tracking, or cluttered backgrounds. To address SORT’s limitations, a series of improved algorithms have emerged. The DeepSORT algorithm [6], building on SORT, introduces deep learning-based appearance modeling. It extracts high-dimensional target features via pre-trained convolutional neural networks, constructs a cost matrix by combining motion similarity and the cosine distance of appearance features, and adopts a cascade matching strategy that prioritizes associating active trajectories. This significantly enhances anti-interference capabilities in scenarios involving occlusion or brief target disappearance. ByteTrack [17] innovatively employs a two-stage matching mechanism: first matching high-confidence detection boxes and then associating low-confidence boxes with unmatched trajectories. This fully leverages all detection information, improving tracking continuity for small targets and in unstable detection scenarios without increasing computational burden. Additionally, Deep OC-SORT [18] enhances the Kalman filter and adopts an IoU-ReID fusion strategy through camera motion compensation, dynamic appearance modeling, and adaptive weighted triplet optimization; BoT-SORT [19] improves tracking robustness in complex dynamic scenes. In 2024, Linh et al. [20] proposed an efficient online tracking framework based on the labeled random finite set (LRFS) theory. By constructing a fuzzy detection model to optimize detection associations in occluded scenes, employing a dynamic appearance feature update mechanism to suppress noise interference, and designing fast filters to handle issues such as target disappearance, reappearance, and long-term occlusion, this framework significantly reduces ID switching and trajectory fragmentation on datasets like MOT17.

Nevertheless, the two-stage multi-target tracking paradigm has a notable drawback: error accumulation. Once a detector produces errors or omissions, the erroneous information is directly propagated to the association stage, severely compromising trajectory integrity. Moreover, maintaining target ID consistency in occluded scenarios remains challenging. Although algorithms like DeepSORT [6] have incorporated pedestrian re-identification technology to enhance association robustness, overall performance still heavily depends on the accuracy of the detection stage.

### 2.2. Joint Detection and Tracking for Multi-Object Tracking

The joint detection and tracking algorithm discards the step-by-step processing mode of the two-stage approach, achieving in-depth collaboration between detection and tracking tasks through an end-to-end architecture. Based on technical implementation paths, it can be categorized into two types: parameter-sharing and fully end-to-end.

The parameter-sharing algorithm shares the feature extraction layer within the backbone network and conducts optimization via joint training, thereby reducing conflicts between different tasks and enhancing the overall performance of the algorithm. For example, the TraDeS model [7] predicts target motion offsets through the Cost Volume Association (CVA) module and fuses temporal features using the Motion-Guided Feature Warping (MFW) module, enabling direct correction of detection results through tracking clues. This approach allows for information exchange between detection and tracking tasks at the feature level. By sharing the feature extraction layer, redundant computations are avoided. Simultaneously, joint training promotes mutual enhancement between the two tasks, collectively improving the algorithm’s performance in complex scenarios.

The fully end-to-end algorithm unifies detection and tracking into a sequence prediction problem and directly outputs the target’s detection results and tracking trajectories by constructing an integrated network structure. These algorithms typically integrate multiple functional modules to achieve a more comprehensive perception of the target. For instance, OffsetNet [8] adopts an end-to-end single-stage multi-task network that integrates detection, segmentation, and tracking functions. Its cross-frame offset prediction and Memory-Enhanced Long Short-Term Self-Attention (MELSA) module can effectively integrate spatiotemporal features, significantly boosting the joint perception ability in scenes involving small targets and cluttered backgrounds. FlowTrack [21] introduces bidirectional optical flow information and periodic consistency checks to strengthen the modeling of motion continuity for fast-moving or small targets and enhances tracking stability in occluded scenes through an error compensation mechanism, effectively overcoming the shortcomings of traditional detection methods in complex motion scenarios.

Additionally, MeMOTR [22], a long-term memory-enhanced Transformer-based multi-target tracker proposed in 2023, significantly improves the tracking continuity of occluded targets by introducing long-term memory vectors and an improved Transformer decoder structure. The independently designed detection decoder reduces semantic misalignment between detection and tracking features, the memory injection mechanism effectively mitigates trajectory interruption, and the adaptive aggregation strategy enhances the tracking robustness of small targets in complex backgrounds. Chen et al. [23] focused on the problem of uneven trajectory distribution in MOT datasets and proposed a long-tail data augmentation method. By synthesizing trajectory data of small targets and occluded scenes to balance the training set distribution, adopting a dynamic loss weight adjustment strategy to enhance the model’s generalization ability to small targets and rare scenes, and using trajectory consistency constraints to reduce trajectory breakage caused by detection noise, this method effectively alleviates performance degradation in small-target and occluded scenes on the MOT20 dataset. The UCMC-Track proposed by Yi et al. [24] addresses the prediction bias caused by camera movement in multi-target tracking through unified camera motion compensation. It estimates camera motion parameters via optical flow and feature matching to achieve global motion estimation, adaptively adjusts the matching range based on camera motion speed, and uses multi-scale feature fusion to improve small object detection accuracy, resulting in a 4.1% improvement in the HOTA index on dynamic scene datasets such as WildTrack.

By enabling in-depth collaboration between detection and tracking tasks, the joint detection and tracking algorithm avoids the problem of accumulated detection errors in the two-stage paradigm. It demonstrates stronger robustness and adaptability in challenging scenarios such as small target tracking, background clutter, and occlusion, opening up new directions for the application of multi-target tracking technology in complex dynamic scenes.

### 2.3. Multi Small-Object Tracking

In recent years, research on MSOT has been continuously advanced, particularly in addressing challenges such as extremely small target sizes, dense distributions, complex backgrounds, and dynamic platforms. A succession of new methods has emerged. For instance, OI-Track, tailored for satellite and drone video scenarios [25], effectively reduces missed detections and identity switches through a trajectory completion module (TCM), an adaptive Kalman filter (AKF), and an iterative extended IoU strategy (I-EIoU), significantly boosting MOTA and IDF1 metrics on the VISO dataset. HGT-Track [26] integrates a heterogeneous image Transformer architecture for visible light and thermal imaging with a target re-detection mechanism, establishing the VT-TinyMOT benchmark for RGB-T multimodal tiny-object tracking tasks and achieving leading performance. DroneMOT, designed for unmanned aerial vehicle platforms, addresses issues like target blurring, size reduction, and camera motion in dynamic aerial photography through a dual-domain integrated attention module, motion-driven association, and an adaptive feature synchronization mechanism, which notably enhances MSOT performance on VisDrone and UAVDT datasets. SiamMOT [27] leverages Siamese networks to implicitly or explicitly integrate motion modeling, enabling efficient online correlation and offering valuable insights for motion estimation of small targets.

### 2.4. Re-Identification for Multiple-Object Tracking

Target re-identification (ReID) technology leverages deep networks to extract global and local features and optimizes the feature space via metric learning. This significantly enhances the capability to distinguish targets across frames and camera shots, providing core support for solving issues such as post-occlusion re-identification and feature confusion caused by background clutter. As a key technology, it plays a vital role in reducing ID switches and trajectory fragmentation. For instance, MITracer [28] has developed an attention mechanism guided by 3D feature volume projection and bird’s-eye view (BEV) to tackle multi-view occlusion problems. By integrating multi-view feature information, it effectively improves the perception and re-identification abilities of occluded targets. Deep IoU [29] addresses challenges like nonlinear motion, severe occlusion, and appearance similarity by expanding the search range through IoU to associate small targets with intense motion. Combining an iterative expansion strategy and two-stage matching of deep appearance features, it enhances tracking performance in scenarios with severe occlusion and dynamic movements without relying on complex motion models. Although it has speed limitations, its approach offers valuable insights for handling extreme cases.

## 3. Methods

### 3.1. Overall Architecture

**Figure 1:**
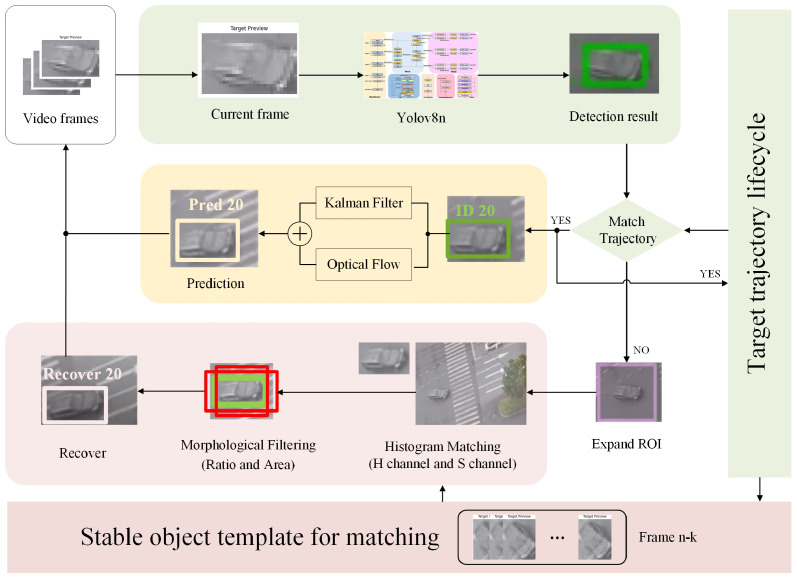
Framework of MOT with ROI expansion and template matching for small-target tracking. The green module performs frame-level target detection, the yellow module accomplishes motion prediction and initial trajectory matching, and the pink module optimizes template recovery to guarantee tracking continuity.

As illustrated in Figure 1, the proposed method builds on the ByteTrack algorithm by retaining its two-stage matching logic (first associating high-confidence detection boxes and then low-confidence ones) while optimizing three core links to address the unique challenges of small-target multi-object tracking (MOT): replacing ByteTrack’s single Kalman motion model with an optical flow-corrected version, adding an adaptive local Region of Interest (ROI) search mechanism for lost targets (not included in ByteTrack), and upgrading the association metric from “motion + simple appearance” to multi-feature fusion. The method takes video frame sequences as input and adopts YOLOv8n as the detection model, with its core framework following a four-step pipeline (each step corresponds to a functional module in Figure 1), and the modules collaborate to tackle issues like fast motion, short-term occlusion, and temporary target loss in small-target MOT scenarios:

1. Initial Target Detection (Green Module in Figure 1): Input video frames are fed into YOLOv8n, which locates small targets (e.g., long-distance pedestrians, small vehicles in aerial imagery) and outputs bounding boxes along with confidence scores. This step lays the foundation for subsequent motion modeling and target association, and its detection results directly determine the initial input quality of the follow-up modules.
2. Optical Flow-Corrected Kalman Filter-Based State Estimation (Yellow Module in Figure 1, Detailed in Section 3.3): To optimize the accuracy of small-target motion state prediction, feature points are first extracted within the detection bounding box, and their optical flow displacement between adjacent frames is computed using the Lucas–Kanade algorithm. These displacement vectors are weighted and fused with the original detection box’s center coordinates to form an enhanced observation value, which is then fed into the Kalman filter. By balancing the long-term stability of the constant-velocity model and responsiveness to instantaneous motion changes, this module integrates Kalman’s global linear prediction with optical flow’s local pixel-level motion capture—effectively reducing prediction lag for fast-moving small targets and improving the update accuracy of motion states.
3. Adaptive Local Search for Lost Target Recovery (Pink Module in Figure 1, Detailed in Section 3.2): When a small target is temporarily lost (i.e., no valid detection match in Step 1, often caused by severe occlusion or motion blur), this mechanism is triggered. Based on the predicted state of the Kalman filter (from Step 2), a local ROI is dynamically generated; meanwhile, the search scale coefficient k is adaptively adjusted according to the target’s historical trajectory velocity and the confidence of its last visible detection. Re-detection is performed only within this ROI—this not only avoids the computational overhead of full-image search but also reduces the risk of missed detections caused by background interference, enabling rapid recovery of lost small targets.
4. Multi-Feature Fusion Target Association (Collaborates with Yellow and Pink Modules in Figure 1, Detailed in Section 3.4): To achieve accurate target matching and consistent ID maintenance for small targets, this module integrates three types of information for multidimensional validation of candidate regions: HSV color histograms (with Bhattacharyya distance as the similarity metric); geometric features (aspect ratio and area constraints); and motion information (Kalman filter predictions from Step 2). Even in complex scenarios involving occlusion or temporary target loss, this multidimensional validation enables reliable target re-identification, further ensuring the continuity of small-target trajectories and the consistency of their identities.

### 3.2. Adaptive Local Search

To enhance the re-identification capability when short-term tracking goals are lost and reduce the computational overhead incurred by full-image searches, this paper proposes an adaptive local search mechanism based on motion prediction. When target tracking fails, this method dynamically generates a Region of Interest according to the predicted state of the Kalman filter and conducts target re-detection within this region, thereby optimizing efficiency while maintaining a high recall rate.

Specifically, in the frame where the target disappears, the system first utilizes the target position in the previous frame to predict the center position, estimated width, and height of the target in the current frame via the Kalman filter. Subsequently, a rectangular candidate search area is constructed. The calculation method for the boundary coordinates of the Region of Interest is as follows:

(1) S=max(a,b)·k

Here, *a* denotes the predicted width of the target in the current frame (output by the Kalman filter), and *b* represents the predicted height of the target in the same frame, also derived from the Kalman filter. The parameter *k* is the search scale coefficient, which is adaptively adjusted: it is set to 1.0 for targets with stable motion and high detection confidence and 1.2–1.5 for fast-moving or low-confidence targets to adapt to different motion and detection scenarios. *S* stands for the side length of the square ROI, calculated by taking the maximum value of the predicted width and height and multiplying it by *k* to ensure the ROI can fully cover the target’s possible position.

(2) $$\left\{\begin{array}{c} x_{1}=\max\left(0,x_{c}-S/2\right)\\ y_{1}=\max\left(0,y_{c}-S/2\right)\\ x_{2}=\min\left(W,x_{1}+S\right)\\ y_{2}=\min\left(H,y_{1}+S\right) \end{array}\right.$$

Among them, $\left(x_{c},y_{c}\right)$ is the predicted center position of the target in the current frame; *W* stands for the width of the entire image frame, and *H* stands for the height of the entire image frame; $x_{1}$ and $y_{1}$ are the coordinates of the top-left corner of the region of interest, and $x_{2}$ and $y_{2}$ are the coordinates of the bottom-right corner of the region of interest; parameter k serves as the search scale coefficient, governing the expansion degree of the search area. A larger value broadens the search scope, favoring the capture of long-distance moving targets but potentially introducing more interference terms. Conversely, a smaller coefficient accelerates search speed but carries the risk of missed detections. Therefore, this paper adaptively adjusts the coefficient based on historical trajectories, incorporating factors like motion speed and detection confidence. This approach minimizes unnecessary computational overhead while preserving high tracking accuracy.

### 3.3. Optical Flow-Corrected Kalman Filter

In traditional MOT frameworks relying on Kalman filtering, target state estimation heavily depends on uniform motion models, which are susceptible to fast motion, occlusion, or nonlinear trajectories—leading to prediction error accumulation. To address this, this paper introduces an optical flow-corrected Kalman filtering approach that enhances responsiveness to instantaneous motion changes while preserving the filter’s long-term stability.

**Figure 2:**
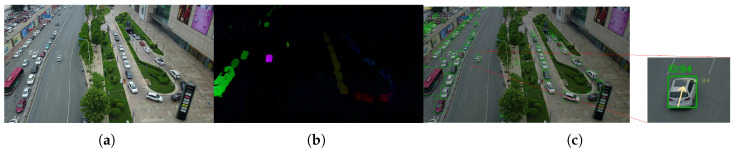
Optical flow prediction of target motion direction. (**a**) Original video frames. (**b**) A schematic diagram of optical flow results: the brighter the color, the higher the motion speed. (**c**) Combining optical flow prediction with MOT, the yellow arrows indicate the direction of motion, with longer arrows corresponding to higher speeds.

Within each frame’s object detection box, several key feature points are extracted, and sparse optical flow algorithms (e.g., Lucas–Kanade) are used to compute their displacements between the current and previous frames. The median of these displacements is subsequently computed to derive the overall optical flow estimation vector. By performing a weighted fusion of this vector with the original detection box’s center coordinates at a specified proportion, as shown in Figure 2, an enhanced measurement closer to the true motion trend is constructed as the observation input for the Kalman filter.

Given the center coordinate of the detection box as $z_{t}={\left[x_{t},y_{t}\right]}^{T}$, the optical flow displacement vector as $d_{t}={\left[\Delta x_{t},\Delta y_{t}\right]}^{T}$, and the fusion weight parameter as α, the optimized measurement value is computed as follows:

(3) $$z_{t}^{\prime }=z_{t}+\alpha \cdot d_{t}=\left[\begin{array}{c} x_{t}+\alpha \Delta x_{t}\\ y_{t}+\alpha \Delta y_{t} \end{array}\right]$$

The original Kalman filter observation equation is expressed as $z_{t}=H\cdot x_{t}+v_{t}$, where $v_{t}$ denotes observation noise terms. Upon introducing optical flow correction, the observation equation is revised to

(4) $$H=\left[\begin{array}{cccc} 1 & 0 & 0 & 0\\ 0 & 1 & 0 & 0 \end{array}\right]$$

(5) $$z_{t}^{\prime }=H\cdot x_{t}+\alpha \cdot d_{t}+v_{t}$$

The state update formula is formalized as

(6) $${\hat{x}}_{t|t}={\hat{x}}_{t|t-1}+K_{t}\cdot \left(z_{t}^{\prime }-H\cdot {\hat{x}}_{t|t-1}\right)$$

Among them, ${\hat{x}}_{t|t-1}$ denotes the predicted state, $K_{t}$ signifies the Kalman gain, and $z_{t}^{\prime }-H\cdot {\hat{x}}_{t|t-1}$ represents the residual obtained from optical flow correction.

This fusion mechanism allows Kalman filtering to retain the long-term stability of the uniform velocity model while simultaneously responding to instantaneous motion changes derived from optical flow. This ensures that state estimation aligns more closely with the target’s instantaneous motion, mitigating prediction lag induced by fast movement or temporary occlusion.

### 3.4. Multi-Feature Fusion Target Association

To enhance the robustness and target persistence of multi-target tracking systems in complex scenarios involving occlusion and missed detections, this paper introduces a multi-feature target association mechanism that integrates appearance and geometric information, following Algorithm 1 as shown. This approach combines color histograms (HSV space), shape features (aspect ratio and area), and motion information (Kalman filter predictions) to enable accurate re-identification and tracking of temporarily occluded targets. While the target maintains a stable ID, the system continuously updates and maintains its appearance template. Unlike the Gibbs sampling approach in LRFS-based methods, which mitigates association ambiguity via probabilistic modeling, this work adopts a “color–geometry–motion” multi-feature hard constraint—this design not only ensures real-time performance but also minimizes mismatches in complex scenarios, making it particularly well-suited for scenes where small targets exhibit sparse feature representations.

In the algorithm, for each tracked target *t* in the current frame *F*, first, the Kalman filter t.KF predicts the bounding box *p*. Then, candidate regions *C* near *p* are extracted. For each candidate region *c* in *C*, histogram similarity *h* (calculated by bhatt_dist between the target’s histogram t.H and the candidate’s histogram calc_hist(c)), aspect ratio *a* (get_ar(c)), geometric similarity *g* (based on the absolute difference of aspect ratios), and motion similarity *m* (based on the distance of centers) are computed. A combined score *s* is obtained by weighted-summing these similarities. Valid matches are stored in *M* if *s* exceeds the threshold $S_{th}$. Finally, for valid matches, the Kalman filter is updated, and the appearance template (histogram t.H and aspect ratio t.A) is updated.
**Algorithm 1** Multi-Feature Fusion Target Association**Require:** Current frame *F*, tracked targets *T***Ensure:** Updated targets $T^{\prime }$
  1:M←{} /* Matches set */  2:**for** t∈T **do**  3:    p←t.KF.predict() /* Predicted bounding box */  4:    C←extract_near(p) /* Candidate regions */  5:    **for** c∈C **do**  6:        h←bhatt_dist(t.H,calc_hist(c)) /* Histogram similarity */  7:        a←get_ar(c) /* Aspect ratio */  8:        g←exp(−|a−t.A|) /* Geometric similarity */  9:        m←exp(−dist(c.c,p.c)) /* Motion similarity */  10:        $s\leftarrow w_{1}\cdot h+w_{2}\cdot g+w_{3}\cdot m$ /* Combined score */  11:        **if** $s>S_{\mathrm{th}}$ **then**  12:           M[t.id]←(c,s) /* Store valid match */  13:        **end if**  14:    **end for**  15:**end for**  16:**for **$t.id,(c^{*},s^{*})\in M$** do**  17:    $t.KF.correct(c^{*}.c)$ /* Kalman filter update */  18:    $t.H\leftarrow \left(1-\alpha \right)\cdot t.H+\alpha \cdot \mathrm{calc}\_\mathrm{hist}\left(c^{*}\right)$ /* Histogram template update */  19:    $t.A\leftarrow \mathrm{get}\_\mathrm{ar}(c^{*})$ /* Aspect ratio update */  20:**end for**  21:**return ***T*

#### 3.4.1. Template Matching

To enhance the system’s adaptability to lighting variations, candidate ROIs are converted from the BGR to the HSV color space, with color histograms constructed using only the H and S channels. Following normalization, the Bhattacharyya distance is employed to quantify the similarity between the template color histogram and the histogram of the candidate region.

(7) $$D=\sqrt{1-\sum_{h,s}\sqrt{{\mathrm{Hist}}_{\mathrm{ref}}\left(h,s\right)\cdot {\mathrm{Hist}}_{i}\left(h,s\right)}}$$

A smaller distance indicates greater feature similarity and a higher matching degree.

#### 3.4.2. Dynamic Shape Constraints and Geometric Filtering

To optimize appearance matching and reduce false matches caused by shape inconsistencies, the system tracks the aspect ratio and bounding-box area of targets in real time and sets geometric consistency thresholds for verification. For targets with stable trajectories, their aspect ratios and areas are recorded. Dynamic thresholds are configured to adapt to rotations and deformations induced by camera motion or changes in target orientation.

After obtaining the ROI, it is converted into a grayscale image, and Gaussian adaptive thresholding is applied to enhance edge features. Then, a 3 × 3 elliptical kernel closing operation is performed to fill holes, connect fragmented regions, remove noise, and preserve the overall shape of the target. Connected regions are extracted from the binary image to obtain candidate contours. The aspect ratio and area of each candidate region are calculated, and regions that meet the aspect ratio and area conditions are selected.

For the candidate regions filtered by geometric constraints, the Bhattacharyya distance between their HSV histograms and the template histogram is calculated in sequence. Candidate boxes with distances below the preset threshold are retained. If there are candidate boxes, the one with the minimum Bhattacharyya distance is selected as the temporary position of the target, which is used to correct the state of the Kalman filter for fine-grained repositioning.

### 3.5. Time Complexity Analysis

The proposed framework mainly consists of four modules: detection, motion estimation, adaptive ROI re-detection, and multi-feature association. The computational complexity of each module is as follows:

- Detection (YOLOv8n): The complexity is approximately O(N·C), where *N* is the number of pixels in the input frame and *C* is the number of convolutional operations. Since modern detectors are GPU-optimized, this stage dominates the total computational cost.
- Kalman Filtering: Both prediction and update steps have complexity $O(d^{2})$, where *d* is the dimension of the state vector (typically <10). This can be considered constant time relative to detection.
- Optical Flow Estimation: Sparse Lucas–Kanade optical flow on *k* feature points has complexity O(k). In practice, k≪N, so the additional overhead is small.
- Data Association: Matching *M* detection boxes with the Hungarian algorithm and multi-feature similarity requires $O(M^{2})$. Since the number of targets per frame is usually moderate, this stage contributes little compared to detection.

Overall, the framework’s time complexity can be expressed as

$$O(N\cdot C+M^{2}+k),$$

which is dominated by the detection stage. The tracking-related operations are lightweight and do not significantly impact runtime.

## 4. Experiments

### 4.1. Datasets

This experiment employed the VisDrone-MOT dataset [30] and the UAVDT dataset [31].

**Figure 3:**
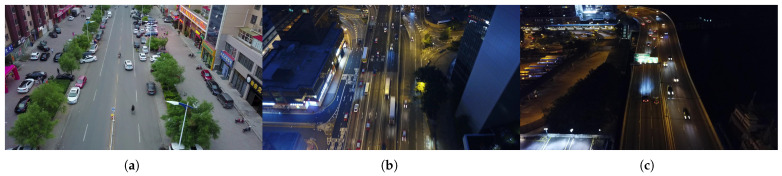
VisDrone-MOT dataset. (**a**) Conventional environment. (**b**) Appearance of motion blur. (**c**) Low-light environment at night.

The VisDrone-MOT dataset, as shown in Figure 3, is a specialized visual benchmark dataset for unmanned aerial vehicle perspectives, constructed by the AISKYEYE team at Tianjin University. It comprises 10,209 static images and 288 videos (with a total of over 260,000 frames), offering 2.6 million meticulously annotated bounding boxes for more than 10 types of targets—including pedestrians and vehicles—across complex scenes such as urban areas, rural regions, and traffic intersections. These targets are often characterized by small sizes, high density, occlusion, and exposure to varying lighting conditions. The annotation attributes encompass occlusion rate, truncation degree, and motion trajectory, supporting five major tasks: object detection, single-target tracking, multi-target tracking, and crowd counting.

**Figure 4:**
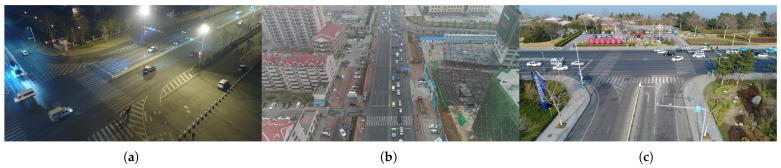
The UAVDT dataset. (**a**) An environment with strong light interference at night. (**b**) Aerial photography from a high-altitude perspective. (**c**) Oblique perspective shooting.

As shown in Figure 4, the UAVDT dataset consists of 100 video sequences captured by drones flying over different urban locations, covering various typical scenes like squares, main roads, and toll stations. The videos have a frame rate of 30 fps and an image resolution of 1080 × 540 pixels, with each frame containing precisely labeled bounding boxes for vehicles. It also provides key attribute information such as vehicle category and occlusion degree and is specifically designed to address the unique perspective and complex environmental challenges associated with drones.

### 4.2. Evaluation Metrics

#### 4.2.1. MOTA

MOTA serves as a core metric for evaluating the overall performance of MOT algorithms. It assesses a tracker’s accuracy by comprehensively penalizing false negatives (FN), false positives (FP), and identity switches (IDSW). The calculation formula is

(8) $$\mathrm{MOTA}=1-\frac{\sum_{i}\left(FN_{i}+FP_{i}+IDSW_{i}\right)}{\sum_{i}GT_{i}}$$

MOTA’s value can theoretically drop to negative infinity, though the closer it gets to 1, the better the tracking performance. This metric emphasizes global sensitivity to detection errors, rooted in the core logic that detection-stage false negatives and false positives impact overall performance far more than tracking-association-stage identity switches (IDSW). Thanks to its intuitiveness and comprehensiveness, MOTA sees widespread use in MOT task evaluation.

#### 4.2.2. False Positives

False positives are a key metric in object detection and multi-object tracking, measuring non-existent targets misidentified or mislocalized by the model due to background misclassification or absent targets. In object detection, an FP refers to a predicted bounding box with insufficient overlap (IoU below the threshold) with any ground-truth target. In multi-target tracking, FPs are categorized into detection-level errors (single-frame false detections) and tracking-level errors (false trajectories from fragmented paths or incorrect associations). FP directly impacts detection accuracy and tracking metrics like MOTA. A high FP rate indicates model overfitting or logical flaws, potentially degrading downstream task reliability.

#### 4.2.3. False Negatives

False negatives are a critical metric in object detection and multi-target tracking, measuring the model’s failure to detect actual targets. It is defined as the count or proportion of ground-truth targets missed by the model. Specifically, in object detection, an FN occurs when a real target is undetected (e.g., no predicted box or IoU below the threshold). In multi-target tracking, FNs are categorized into detection-level misses (single-frame non-detections) and tracking-level losses (target disappearances due to trajectory breaks or ID-switch errors).

FN directly impacts recall rates and tracking metrics like MOTA. A high FN rate indicates insufficient detection sensitivity or tracking association failures, potentially causing key target omissions in downstream tasks (e.g., security monitoring). Optimization strategies include lowering detection confidence thresholds, enhancing feature learning for complex scenes, and improving trajectory association robustness.

#### 4.2.4. HOTA

HOTA is a comprehensive metric for multi-target tracking performance, designed to address limitations of traditional metrics like MOTA and IDF1 in balancing detection, association, and localization. Its core design combines detection accuracy (DetA), association consistency (AssA), and localization precision (LocA) into a single score via high-order correlation analysis. By using geometric averaging across different localization thresholds, HOTA holistically reflects tracking system performance.

HOTA’s unique advantage lies in its decomposability: it breaks down into sub-metrics like detection recall and association accuracy, quantifying five key errors—missed detections, false positives, identity switches, and trajectory breaks—to enable fine-grained model diagnosis. Unlike traditional metrics focusing on adjacent-frame matching, HOTA emphasizes long-term cross-frame association. By balancing detection and association weights, it reduces single-task bias, aligning more closely with human visual evaluation in complex scenarios (e.g., occlusion, appearance similarity).

#### 4.2.5. IDF1

IDF1 is a key metric for multi-target tracking, balancing detection accuracy and identity consistency. It takes the harmonic mean of detection recall (capturing ground-truth targets) and identity precision (assigning correct labels), assessing a tracker’s ability to detect targets and keep identities accurate. Vital for long-term tracking (e.g., in crowds), high IDF1 indicates few false detections/identities or identity switches. Optimizing it requires boosting detection to cut errors and improving association to preserve identities.

### 4.3. Hyperparameter Experiments

In the detector section, YOLOv8n was uniformly adopted to train part of the data from VisDrone MOT, with the remaining untrained data used for testing. In the matching section, for a single matching algorithm, the detection confidence threshold is set to 0.5. For the secondary matching algorithm, the first matching reliability threshold is set to 0.5, and the second one to 0.1. The maximum tracking loss frame count is set to 150 frames. Local detection expands the search area to twice the target size and continues detection for 30 frames after the target is lost.

To determine the optimal Bhattacharyya distance threshold for multi-feature correlation, this paper evaluates system performance at thresholds of 0.5, 0.4, 0.3, and 0.2. The results are presented in Table 1.

When the threshold is set high (0.5), FN remains low while FP surges, as the system shows low tolerance for mismatches—hindering target recovery. Notably, thresholds exceeding 0.5 were not selected for further analysis, as practical tests revealed even more severe issues: such values drastically relax the similarity requirement for feature matching, causing FP to escalate exponentially. This leads to a proliferation of false trajectories (e.g., misidentifying background noise or irrelevant objects as targets) and a sharp decline in core metrics like MOTA and HOTA, rendering the results practically meaningless for robust tracking. At a threshold of 0.3, overall performance peaks: FN and FP strike a balance, with both HOTA and MOTA reaching their highest values, marking the optimal inflection point for detection robustness and accuracy. When the threshold drops further to 0.2, although FP decreases, FN rises sharply, causing system overfitting that amplifies the risk of missed detections.

### 4.4. Comparison to State-of-the-Art Methods

To validate the superior performance of our method, six MOT methods are chosen, including ByteTrack [17], BotSORT [19], OC-SORT [32], DeepOCSORT [18], BoostTrack [33], and Imprassoc.

#### 4.4.1. Qualitative Analysis

**Figure 5:**
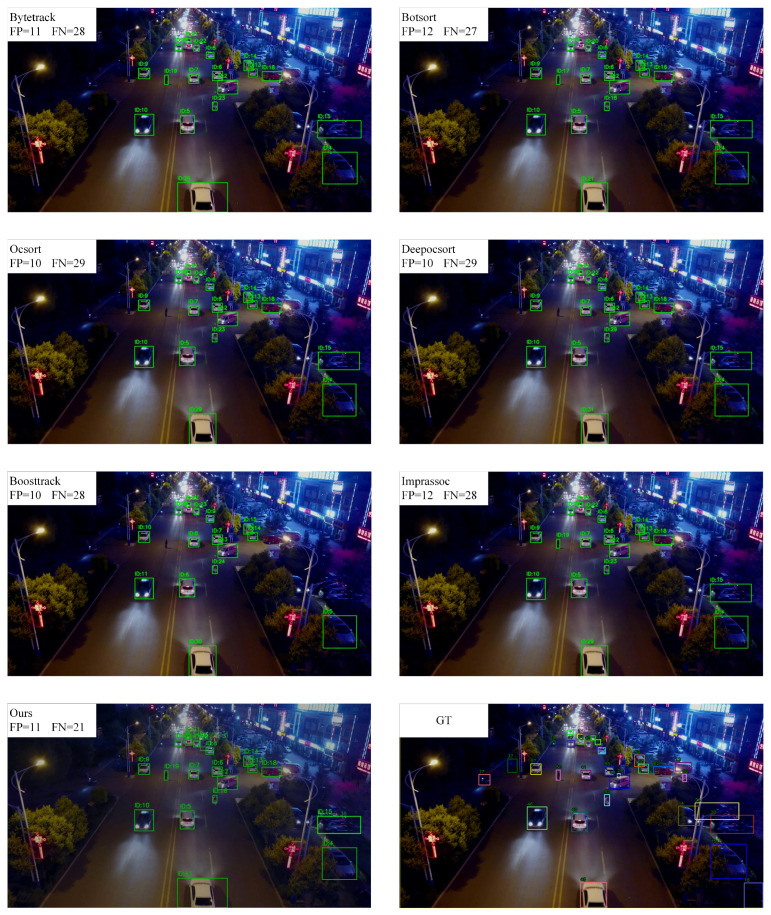
The detection results of six algorithms. It presents the comparison of detection outcomes from MOT algorithms (ByteTrack, BotSort, OCSort, DeepOCSort, BoostTrack, Imprassoc, and ours). Each algorithm’s result is accompanied by counts of FP and FN. The GT provides the reference standard for accurate object detection.

**Figure 6:**
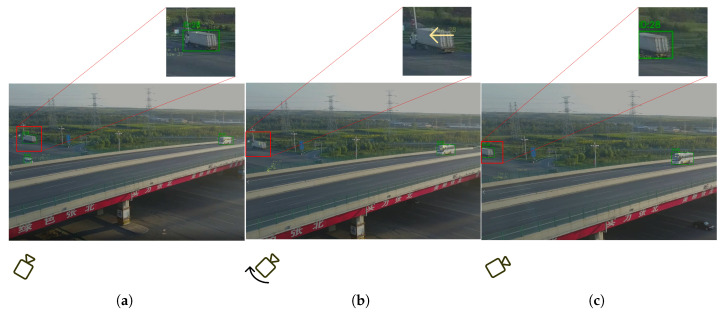
Effect of optical flow compensation. (**a**) Initial position of camera. (**b**) Rapid rotation of camera leads to target loss, which is tracked via optical flow (yellow arrow). (**c**) Target is re-detected to facilitate subsequent tracking.

As shown in Figure 5, in the context of ID stability performance within MOT tasks, algorithms like ByteTrack face certain performance drawbacks when pitted against traditional appearance matching-based methods and deep-learning methods typified by DeepOCSORT. Take the frame in the figure as an example, where the camera is in rapid motion and there is partial occlusion from roadside trees. These conditions make it a struggle to continuously and stably link the trajectories of the same target. To tackle this problem, as shown in the pink box in Figure 6, when a target is temporarily lost, this paper does not have to depend on deep-learning models for secondary detection and feature matching to retrieve the target. Simultaneously, in the aspect of processing speed, since time-consuming procedures such as feature extraction and similarity calculation in deep-learning methods are bypassed, its operational efficiency has been notably enhanced compared with deep learning-based solutions. This makes it a better fit for scenarios with high real-time demands.

#### 4.4.2. Quantitative Analysis

The performance evaluation on the VisDrone-MOT dataset, as presented in Table 2, underscores the superiority of our proposed method. It surpasses all baseline algorithms across key metrics: achieving an MOTA of 63.24 and an IDF1 of 59.41. These values represent improvements of 22.68 and 5.01, respectively, over DeepOCSort, which ranks second in both MOTA and IDF1. Notably, the FP + FN count is drastically reduced from 191,693 (the highest among baselines, exhibited by BoostTrack) to 89,102. This reduction underscores the method’s enhanced capability to maintain target detection accuracy in complex scenes. With 1046 Mostly Tracked (MT) targets (the highest across all methods) and only 292 Mostly Lost (ML) targets (the lowest), the system excels at sustained trajectory maintenance and minimizing target loss. Although its HOTA of 34.84 is slightly lower than that of BotSort and DeepOCSort, the comprehensive performance in terms of MOTA, FP + FN, MT, and IDF1 demonstrates that our method effectively balances tracking precision, trajectory continuity, and identity recognition. Thus, it shows strong applicability in multi-object tracking tasks.

Results on the UAVDT dataset, as presented in the Table 3, further validate our approach. Leading with MOTA = 63.24 and IDF1 = 59.41, our method maintains high accuracy. While ByteTrack and OCSort exhibit marginal speed advantages (FPS of 20.19 and 20.44, respectively), our method, with an FPS of 9.88, still demonstrates competitiveness in performance–efficiency balance. BotSort and DeepOCSort show relatively high HOTA (39.10 and 38.80), yet they lag behind our method in MOTA and IDF1. BoostTrack, with the highest FP + FN count of 191,693, frequently suffers from trajectory fragmentation or target missing in complex scenarios. In contrast, our method, with the lowest FP + FN count of 89,102 and the highest MT (1046) along with the lowest ML (292), demonstrates remarkable stability, ensuring reliable tracking performance even under challenging conditions.

### 4.5. Ablation Experiments

To evaluate the performance contribution of each core module, this paper designs four ablation comparisons based on the ByteTrack framework using the VisDrone-MOT dataset. The results are presented in Table 4.

The results showed that introducing the local detection mechanism (ROI) improved MOTA from 40.56 to 60.31, a rise of nearly 20 percentage points, and reduced FP + FN count from 162,538 to 96,868. This indicates that this module significantly enhances the recovery of occluded or missed targets. However, it also caused changes in other metrics (e.g., HOTA slightly decreased from 35.50 to 33.90), suggesting that regional detection occasionally introduces mismatched targets, which may impact some performance aspects. Introducing the optical flow correction mechanism (optical flow) also significantly boosted overall performance. IDF1 rose from 56.14 to 59.45, evidence that accurate target motion trend modeling improves ID consistency. As shown in Figure 6, when the target is lost due to image blurring caused by rapid camera movement, optical flow can still achieve a certain degree of tracking and prediction. Combining both (ROI + optical flow) achieved optimal results in key metrics like MOTA (reaching 63.24), MT (increasing to 1046), etc. FP + FN further decreased to 89,102, demonstrating that their synergistic effect balances target recovery, motion prediction, and appearance modeling to maximize system performance, though FPS showed a reduction compared to some single-mechanism cases.

### 4.6. Computational Efficiency Evaluation

To evaluate computational efficiency, We benchmarked our method on an NVIDIA RTX 1080ti GPU (manufactured by NVIDIA, Santa Clara, California, USA), an Intel i7-6850K CPU (manufactured by Intel, Santa Clara, California, USA), and 32 GB RAM. We measured the average single-frame processing time across the UAVDT datasets and compared it with representative baselines (ByteTrack and DeepOCSORT). Specifically, ByteTrack achieved an average single-frame processing time of 42.2 ms, DeepOCSORT took 65.8 ms, and our proposed method took 48.8 ms. According to the results, the ByteTrack method stands out with the optimal computational efficiency, recording an average single-frame processing time of only 42.2 ms. This excellent performance enables ByteTrack to process nearly 24 frames per second, fully meeting the real-time requirements of most high-demand MOT scenarios. Our proposed method achieves an average single-frame processing time of 48.8 ms, which is approximately 15.6% longer than ByteTrack. Despite this gap, it still maintains good computational efficiency (supporting about 20.5 frames per second) and outperforms the DeepOCSORT method significantly. DeepOCSORT shows the lowest computational efficiency among the three, with an average single-frame processing time reaching 65.8 ms—38% slower than our method and 55.9% slower than ByteTrack. This longer processing time may limit its application in scenarios where low latency is a key constraint.

## 5. Conclusions

This paper presents a dual motion modeling framework integrating Kalman filtering and optical flow features to address key challenges in MSOT, such as occlusion recovery, trajectory continuity, and detection accuracy. The framework incorporates an adaptive local re-detection mechanism and a multi-feature fusion target association strategy to systematically enhance MOT system robustness and accuracy in complex scenes.

Specifically, this work introduces three key modules based on the lightweight detector YOLOv8n and the ByteTrack framework:

1. An optical flow-corrected Kalman filter that enhances state prediction responsiveness and timeliness, effectively mitigating prediction lag caused by rapid motion or occlusion.
2. An adaptive local search mechanism that improves post-loss re-detection success rates and significantly reduces missed detections.
3. A multi-feature object association mechanism integrating color histograms, geometric features, and motion trajectories to strengthen identity consistency and matching robustness under occlusion.

Extensive comparative and ablation experiments on two small-target datasets (VisDrone-MOT, UAVDT) confirm our method’s significant advantages in core metrics (MOTA, IDF1, HOTA). Notably, hyperparameter experiments (Table 1) further verify the critical role of the Bhattacharyya distance threshold in multi-feature correlation: setting it to 0.3 yields optimal performance balance (MOTA: 63.24, HOTA: 34.84), with false positives (FP: 12,348) and false negatives (FN: 76,754) kept reasonable. A too-high threshold (e.g., 0.5) causes FP surges due to low mismatch tolerance, while a too-low one (e.g., 0.2) triggers overfitting and higher missed detection risks—this optimal parameter supports stable system operation in complex scenarios. Against state-of-the-art (SOTA) methods, our approach shows strong competitiveness: On VisDrone-MOT, it reaches MOTA 63.24 (22.68 higher than the second-ranked DeepOCSort) and IDF1 59.41 (5.01 higher than DeepOCSort), with the lowest FP+FN (89,102, drastically lower than BoostTrack’s 191,693) and highest Mostly Tracked (MT: 1046) targets. On UAVDT, it maintains a leading MOTA of 61.4 and IDF1 of 66.1, with the fewest Mostly Lost (ML: 56) targets. It excels in scenarios with frequent occlusion, dense targets, and low detector confidence, delivering superior target retention and trajectory stability. Meanwhile, it preserves good real-time performance (20.5 FPS on UAVDT, 9.88 FPS on VisDrone-MOT) while improving accuracy, showing strong engineering application potential

Although this approach advances small-target tracking, areas for improvement include the following:

- Current sparse optical flow estimation struggles with complex backgrounds or textureless targets; future work could integrate deep learning-based dense optical flow networks.
- The multi-feature fusion strategy introduces computational overhead during re-detection; optimizing feature extraction/matching efficiency is critical for large-scale real-time deployment.
- Cross-category tracking in open scenarios remains challenging; future research could incorporate open-vocabulary recognition and semantic modeling for more intelligent, generalized tracking.

In summary, the proposed multi-module collaborative framework offers strong practicality and scalability for MSOT tasks, providing novel insights and technical pathways for multi-target tracking in complex environments.

## Figures and Tables

**Table 1:** Results of hyperparameter experiments.

Thresholds	MOTA ↑	HOTA ↑	FP ↓	FN ↓	IDF1 ↑	FPS ↑
0.5	49.60	29.92	74,879	**42,122**	59.43	**8.66**
0.4	59.70	31.91	32,178	60,104	**60.33**	9.25
**0.3**	**63.24**	**34.84**	12,348	76,754	59.41	9.88
0.2	60.88	34.45	**9535**	87,938	58.66	9.90

↑ Indicates that a higher value is preferable; ↓ indicates that a lower value is preferable. The values in bold represent the optimal ones.

**Table 2:** Results of VisDrone-MOT comparative experiments.

Method	MOTA ↑	HOTA ↑	FP + FN ↓	MT ↑	PT	ML ↓	IDF1 ↑	FPS ↑
ByteTrack	40.56	35.50	162,538	425	674	864	54.45	20.19
BotSort	37.78	**39.10**	171,315	414	570	979	53.86	10.59
OCSort	31.99	33.50	186,283	275	653	1035	46.72	**20.44**
DeepOCSort	41.86	38.80	158,575	429	728	806	56.76	9.80
BoostTrack	30.25	31.60	191,693	245	623	1095	45.66	9.16
Imprassoc	52.17	36.80	123,955	757	733	527	52.24	9.59
**Ours**	**63.24**	34.84	**89,102**	**1046**	625	**292**	**59.41**	9.88

↑ Indicates that a higher value is preferable; ↓ indicates that a lower value is preferable. The values in bold represent the optimal ones.

**Table 3:** Results of UAVDT comparative experiments.

Method	MOTA ↑	HOTA ↑	FP + FN ↓	MT ↑	PT	ML ↓	IDF1 ↑	FPS ↑
ByteTrack	58.6	44.45	111,954	482	256	108	66	23.7
BotSort	57.8	**45.4**	115,275	432	263	149	**67**	15.4
OCSort	54.5	42.71	122,535	340	304	202	59.5	**24.1**
DeepOCSort	58.4	43.22	110,821	443	281	122	62.6	15.2
BoostTrack	45.6	39.21	145,095	286	360	200	57.6	12.3
Imprassoc	56.6	40.67	113,770	550	229	67	56.8	15.2
**Ours**	**61.4**	44.64	**101,908**	**561**	229	**56**	66.1	20.5

↑ Indicates that a higher value is preferable; ↓ indicates that a lower value is preferable. The values in bold represent the optimal ones.

**Table 4:** Results of ablation experiments.

Method	MOTA ↑	HOTA ↑	FP + FN ↓	MT ↑	PT	ML ↓	IDF1 ↑	FPS ↑
base	40.56	**35.50**	162,538	425	674	864	56.14	**20.19**
ROI	60.31	33.90	96,868	981	572	301	**59.80**	10.29
optical flow	57.91	34.54	106,549	911	694	359	59.45	14.07
**ROI + optical flow**	**63.24**	34.84	**89,102**	**1046**	625	**292**	59.41	9.88

↑ Indicates that a higher value is preferable; ↓ indicates that a lower value is preferable. The values in bold represent the optimal ones.

## Data Availability

Data are contained within the article.
